# Comprehensive proteomics data on whole rice grain of selected pigmented and non-pigmented rice varieties using SWATH-MS approach

**DOI:** 10.1016/j.dib.2020.105927

**Published:** 2020-06-26

**Authors:** Yun Shin Sew, Wan Mohd Aizat, Mohd Shahril Firdaus Ab Razak, Rabiatul-Adawiah Zainal-Abidin, Sanimah Simoh, Norliza Abu-Bakar

**Affiliations:** a Malaysian Agricultural Research & Development Institute (MARDI), 43300 Serdang, Selangor, Malaysia; b Institute of Systems Biology (INBIOSIS), Universiti Kebangsaan Malaysia (UKM), 43600 UKM Bangi, Selangor*,* Malaysia

**Keywords:** Proteomics, Pigmented rice, Non-pigmented rice, Nutritional traits, SWATH-MS

## Abstract

The proteome data of whole rice grain is considerably limited particularly for rice with pigmentations such as black and red rice. Hence, we performed proteome analysis of two black rice varieties (BALI and Pulut Hitam 9), two red rice varieties (MRM16 and MRQ100) and two white rice varieties (MR297 and MRQ76) using label-free liquid chromatography Triple TOF 6600 tandem mass spectrometry (LC-MS/MS). Our aim was to profile and identify proteins related to nutritional (i.e. antioxidant, folate and low glycaemic index) and quality (i.e. aromatic) traits based on peptide-centric scoring from the Sequential Window Acquisition of All Theoretical Mass Spectra (SWATH-MS) approach. Both information dependent acquisition (IDA) and SWATH-MS run were performed in this analysis. Raw data was then processed using ProteinPilot software to identify and compare proteins from the six different varieties. In future, this proteomics data will be integrated with previously obtained genomics [1] and transcriptomics [2] data focusing on the above nutritional and quality traits, with an ultimate aim to develop a panel of functional biomarkers related to those traits for future rice breeding programme. The raw MS data of the pigmented and non-pigmented rice varieties have been deposited to ProteomeXchange database with accession number PXD018338.

Specifications table

**Table:** 

Subject	Plant Biology
Specific subject area	Proteomics and Plant Sciences
Type of data	LC-MS/MS spectral data
How data were acquired	MS analysis using Eksigent NanoLC^Ⓡ^ Ultra systems (Eksigent Technologies, Dublin, CA, USA) coupled to Triple TOF 6600 mass spectrometer (AB SCIEX Foster City, CA, USA)
Data format	Raw (.wiff & .wiff.scan) and processed (.group)
Parameters for data collection	The matured rice seeds of Malaysian rice varieties with varying pigmentations known as black rice (BALI and Pulut Hitam 9), red rice (MRM16 and MRQ100) and white rice (MR297 and MRQ76) were collected from MARDI rice field plots. The pigmented rice (BALI, Pulut Hitam 9, MRM16 and MRQ100) were chosen due to their high antioxidant properties [3]. Among the two non-pigmented rice varieties, MRQ76 is aromatic rice [4] and MR297 is known for high micronutrients [3]. Rice seeds were dehusked and total proteins were extracted from whole rice grains using modified phenol extraction method as described in Faurobert et al. (2007) [5].
Description of data collection	The extracted total proteins were reduced with dithiothreitol, alkylated with iodoacetamide, digested with trypsin and purified before subjected to LC-MS/MS run using both information-dependent acquisition (IDA) experiments and SWATH-MS analysis.
Data source location	City/Town/Region: Serdang, Selangor Country: Malaysia Latitude and longitude (and GPS coordinates) for collected samples/data:] 2.9885871″N 101.697955417″E
Data accessibility	Proteome data of pigmented (BALI, PH9, MRM16 and MRQ100) and non-pigmented (MR297 and MRQ76) rice varieties are accessible via ProteomeXchange with identifier PXD018338 (https://www.ebi.ac.uk/pride/archive/projects/PXD018338).

## Value of the data

• This dataset represents a protein catalogue for data mining and identification of potential protein biomarkers related to important grain traits in Malaysian rice varieties.
• Researchers and breeders may use this data to make informed decision on breeding programs for certain selected rice traits.
• The pigmented and non-pigmented rice proteome data provide additional information apart from the genomics and genetics data of pigmented and non-pigmented Malaysian rice varieties.
• The dataset allows integrative and complementary approach with other omics datasets including genomics and transcriptomics. The data contributes to System Biology approaches for deciphering informations related to important grain traits in Malaysian rice varieties
• Integration between protein biomarkers and previously identified molecular markers such as single nucleotide polymorphisms can be developed into high-confident biomarkers to facilitate the genetic improvement of rice varieties with enhanced nutritional and quality traits.
• The data obtained provide additional informations for the identification of potential biomarkers and DNA markers to assist future rice breeding programme.

## 1 Data description

The proteome of four Malaysian pigmented (BALI, Pulut Hitam 9, MRM16 and MRQ100) and two non-pigmented rice varieties (MR297 and MRQ76) were profiled in this analysis. These pigmented and non-pigmented rice varieties were Indica rice subspecies (*Oryza sativa* L. ssp. indica). All the six varieties, with three biological replicates each, were subjected to SWATH-MS and information-dependent acquisition (IDA) analyses to generate respective raw files (.wiff and .scan.wiff). All 18 samples (six varieties with three biological replicates) were run individually in the SWATH-MS run. Whereas for the IDA runs, 17 fractions were first obtained from pooled samples using High pH reverse phase-high performace liquid chromatography (HpH-RP‐HPLC) before analysed in the LC-MS/MS system. The IDA datasets were then used as spectral library (.group files) for the subsequent analysis of SWATH-MS quantitative data. These MS datasets were deposited to the ProteomeXchange Consortium (http://proteomecentral.proteomexchange.org) via the PRIDE [6] partner repository, with the dataset identifier PXD018338 (https://www.ebi.ac.uk/pride/archive/projects/PXD018338).

## 2 Experimental design, materials, and methods

### 2.1 Plant materials and total protein extraction

Mature seeds of each pigmented (BALI, Pulut Hitam 9, MRM16 and MRQ100) and non-pigmented (MR297 and MRQ76) rice varieties were obtained in the field plots at MARDI Seberang Perai, Penang, Malaysia. The seeds were dehusked and the whole rice grain tissue (three plants of each variety) were ground into fine powder using liquid nitrogen. Total proteins were extracted based on a modified phenol extraction method as described in Faurobert et al. (2007) [5] with modification. Approximately 0.5 g fine powder was suspended in 3 vol of extraction buffer (containing 700 mM sucrose, 500 mM Tris pH 8, 100 mM KCl, 2% (v/v) β-mercaptoethanol, 2 mM phenylmethylsulfonyl fluoride pH 8.5) and incubated for 10 min on ice. Afterward, an equal volume of Tris-saturated phenol pH 8.0 was added. The mixtures were then shaken for 10 min at room temperature and centrifuged at 13,000 *x g* for 10 min at 4 °C to separate the phenolic and aqueous phases. The phenolic phase was recovered and re-extracted with the same volume of extraction buffer. After the phenol extraction, proteins were precipitated using a precipitation solution (0.1 M ammonium sulphate in cold methanol) at −20 °C overnight. After centrifugation for 10 min (13,000 x g, 4 °C), the protein pellet was washed three times with the precipitation solution and once with chilled acetone. The resulting protein pellets were air-dried and sent to The Australian Proteome Analysis Facility (APAF), Macquarie University, Sydney NSW, Australia.

### 2.2 Sample preparation

The acetone precipitated protein pellets were resuspended in 8 M urea in 100 mM Tris‐HCl (pH 8.8), vortexed and water‐sonicated for 15 min. The protein concentrations were then determined by bicinchoninic acid (BCA) assay. Approximately 50 µg of each sample (all 18 samples) was taken and diluted with 100 mM triethyl ammonium bicarbonate (TEAB). Samples were subsequently reduced with 10 mM dithiothreitol (DTT) and alkylated with 25 mM iodoacetamide (IAA) before subjected to a 16 h trypsin digestion at 37 °C. The digested samples were purified, dried and resuspended in 50 µL of loading buffer (2% acetonitrile and 0.1% formic acid).

Peptide samples were subjected to peptide separation using Eksigent NanoLC^Ⓡ^ Ultra systems (Eksigent Technologies, Dublin, CA, USA) equipped with analytical column nano cHiPLC column (15 cm x 200 µm, 120 Å, 3 μm) coupled to TripleTOF™ 6600 mass spectrometer (AB SCIEX, USA). For SWATH analysis, 4 µL for each sample was diluted with 6 µL of loading buffer and SWATH were acquired in random order with one blank run after each sample. Furthermore, ion libraries were generated by pooling 12.5 µL of each sample (total 18 samples) before fractionation by High pH RP‐HPLC (HpH-RP-HPLC). The pooled sample was purified, dried and resuspended in mobile phase buffer A (5 mM ammonium hydroxide solution, pH 10.5). After sample loading and washing with 3% buffer B (5 mM ammonia solution with 90% acetonitrile, pH 10.5) for 10 min at a flow rate of 300 μL/min, the buffer B concentration was increased from 3% to 30% over 55 min and then to 70% between 55 and 65 min and to 90% between 65–70 min. The eluent was collected every 2 min at the beginning of the gradient and at one minute intervals for the rest of the gradient. Upon HpH‐RP‐HPLC separation, 17 fractions were concatenated (0–85 min), dried and resuspended in 11 µL of loading buffer. Subsequently, 10 µL per fraction was taken for 2D-IDA analysis.

### 2.3 Data acquisition

For analysis in 2D-IDA, 10 µL of sample was first injected onto a reverse‐phase C18 self‐packed peptide trap for pre‐concentration and desalted with loading buffer, at 5 µL/min for 3 min before switching to the analytical column. Peptides were eluted from the column using a linear solvent gradient from mobile phase A: mobile phase B (95:5) to mobile phase A: mobile phase B (65:35) at 600 nL/min over a period of 120 min. After peptide elution, the column was cleaned with 95% buffer B for 6 min and then equilibrated with 95% buffer A for 10 min before the injection of next sample. The reverse phase nanoLC eluent was subjected to positive ion nanoflow electrospray analysis in an information dependant acquisition mode (IDA). In the IDA mode, a Time-of-flight mass spectrometry (TOF-MS) survey scan was acquired (350–1500 *m/z*, 0.25 s) with the 20 most intense multiply charged ions (2+ ‐ 5+; exceeding 200 counts per sec) in the survey scan sequentially subjected to MS/MS analysis. MS/MS spectra were accumulated for 100 msec in the mass range of 100–1800 *m/z* with rolling collision energy.

For data independent acquisition using SWATH method, 10 µL sample was injected onto a reverse‐phase C18 self‐packed peptide trap for pre‐concentration and desalted with loading buffer, at 5 µL/min for 3 min. The peptide trap was then switched into line with the analytical column. Peptides were eluted from the column using a linear solvent gradient from mobile phase A: mobile phase B (95:5) to mobile phase A: mobile phase B (65:35) at 600 nL/min over a 120 min period. After peptide elution, the column was cleaned with 95% buffer B for 6 min and then equilibrated with 95% buffer A for 10 min before next sample injection. The reverse phase nanoLC eluent was subjected to positive ion nanoflow electrospray analysis in a data independent acquisition (SWATH). In SWATH mode, first a TOFMS survey scan was acquired (350–1500 *m/z*, 50 msec) then the 100 predefined *m/z* ranges were sequentially subjected to MS/MS analysis. MS/MS spectra were accumulated for 30 ms in the mass range of 350–1500 *m/z* with rolling collision energy optimised for *m/z* in *m/z* window +10%.

### 2.4 Data processing

The data files generated by 2D‐IDA‐MS analysis were searched with ProteinPilot version 5.0 (AB SCIEX Foster City, CA, USA) using the Paragon™ algorithm in thorough mode. Carbamidomethylation of cystein residues was selected as a fixed modification. An Unused Score cut‐off was set to 1.3 (95% confidence for identification), and global protein false discovery rate of 1%. Two rice databases which are available in UniProt\Proteomes were combined and used for protein annotation, 1. *Oryza sativa* ssp. japonica (Strain: cv. Nipponbare, Proteome ID: UP000059680) and 2. *Oryza sativa* ssp. indica (Strain: cv. 93–11, Proteome ID: UP000007015). The ion library was constructed from 2D‐IDAs. Ion library and SWATH data files were imported into PeakView version 2.2 (AB SCIEX Foster City, CA, USA. Protein peak area information in SWATH data were extracted using PeakView software with the following parameters: Top 6 most intense fragments of each peptide were extracted from the SWATH data sets (75 ppm mass tolerance, 5 min retention time window). Shared and modified peptides were excluded. After data processing, peptides (max. 100 peptides per protein) with confidence ≥ 99% and FDR ≤ 1% (based on chromatographic feature after fragment extraction) were used for quantitation.

## Declaration of Competing Interest

The authors declare that they have no known competing financial interests or personal relationships that could have appeared to influence the work reported in this paper.

## Appendix Supplementary materials

Image, application 1

## References

[1] Adawiah Zainal R., Abu Bakar N., Sew Y.S., Simoh S., Mohamed-Hussein Z.A. (2019). Discovery of Functional SNPs via Genome-wide exploration of Malaysian pigmented rice. Int. J. Genomics.

[2] Adawiah Zainal R., Zainal Z., Mohamed Hussein Z.A., Abu-Bakar N., Ab Razak M.S.F., Simoh S., Sew Y.S. (2020). RNA-seq data from whole rice grains of pigmented and non-pigmented Malaysian rice varieties. Data in Brief.

[3] Sew Y.S., Ahmad M.A., Abd Rashid M.R., Abu Bakar N., Machap C., Ling A.C.K., Zainal Abidin R.A., Rozano L., Simoh S. (2016). Antioxidant activities and microelement composition of Malaysian local pigmented and non-pigmented rice varieties. Trans. Persatuan Genet. Malays..

[4] Harun R., Halim N.A., Engku Ariff E.E., Serin T. (2018). Consumer preferences on Malaysia's specialty rice. FFTC Agric. Policy Platf. (FFTC-AP).

[5] Faurobert M., Mihr C., Bertin N., Pawlowski T., Negroni L., Sommerer N., Causse M. (2007). Major proteome variations associated with cherry tomato pericarp development and ripening. Plant Physiol.

[6] Vizcaíno J.A., Csordas A., Del-Toro N., Dianes J.A., Griss J., Lavidas I., Mayer G., Perez-Riverol Y., Reisinger F., Ternent T., Xu Q.W. (2016). pdate of the PRIDE database and its related tools.. Nucleic Acids Res.

